# By Default: The Effect of Prepopulated Prescription Quantities on Opioid Prescribing in the Emergency Department

**DOI:** 10.5811/westjem.2017.10.33798

**Published:** 2018-02-12

**Authors:** Jamie R. Santistevan, Brian R. Sharp, Azita G. Hamedani, Scott Fruhan, Andrew W. Lee, Brian W. Patterson

**Affiliations:** * University of Wisconsin School of Medicine and Public Health, BerbeeWalsh Department of Emergency Medicine, Madison, Wisconsin; † University of California San Francisco, Zuckerberg San Francisco General; ‡ Hospital, Department of Emergency Medicine, San Francisco, California; § Kaiser Permanente Oakland Medical Center, Emergency Department, Oakland, California; ¶ Health Innovation Program, University of Wisconsin-Madison, Madison, Wisconsin

## Abstract

**Introduction:**

Opioid prescribing patterns have come under increasing scrutiny with the recent rise in opioid prescriptions, opioid misuse and abuse, and opioid-related adverse events. To date, there have been limited studies on the effect of default tablet quantities as part of emergency department (ED) electronic order entry. Our goal was to evaluate opioid prescribing patterns before and after the removal of a default quantity of 20 tablets from ED electronic order entry.

**Methods:**

We performed a retrospective observational study at a single academic, urban ED with 58,000 annual visits. We identified all adult patients (18 years or older) seen in the ED and discharged home with prescriptions for tablet forms of hydrocodone and oxycodone (including mixed formulations with acetaminophen). We compared the quantity of tablets prescribed per opioid prescription 12 months before and 10 months after the electronic order-entry prescription default quantity of 20 tablets was removed and replaced with no default quantity. No specific messaging was given to providers, to avoid influencing prescribing patterns. We used two-sample Wilcoxon rank-sum test, two-sample test of proportions, and Pearson’s chi-squared tests where appropriate for statistical analysis.

**Results:**

A total of 4,104 adult patients received discharge prescriptions for opioids in the pre-intervention period (151.6 prescriptions per 1,000 discharged adult patients), and 2,464 post-intervention (106.69 prescriptions per 1,000 discharged adult patients). The median quantity of opioid tablets prescribed decreased from 20 (interquartile ration [IQR] 10–20) to 15 (IQR 10–20) (p<0.0001) after removal of the default quantity. While the most frequent quantity of tablets received in both groups was 20 tablets, the proportion of patients who received prescriptions on discharge that contained 20 tablets decreased from 0.5 (95% confidence interval [CI] [0.48–0.52]) to 0.23 (95% CI [0.21–0.24]) (p<0.001) after default quantity removal.

**Conclusion:**

Although the median number of tablets differed significantly before and after the intervention, the clinical significance of this is unclear. An observed wider distribution of the quantity of tablets prescribed after removal of the default quantity of 20 may reflect more appropriate prescribing patterns (i.e., less severe indications receiving fewer tabs and more severe indications receiving more). A default value of 20 tablets for opioid prescriptions may be an example of the electronic medical record’s ability to reduce practice variability in medication orders actually counteracting optimal patient care.

## INTRODUCTION

Painful conditions make up 42% of all emergency department (ED) visits.^1^ With the increasing focus on analgesia by The Joint Commission’s pain management standards and emphasis on analgesia in patient satisfaction surveys, it is no surprise that medical use of opioids and opioid analgesic prescriptions has been increasing since the early 1990s.^2^–^8^ Unfortunately, there has also been an increase in prescription opioid abuse and misuse, with a rise in opioid-related events including increases in opioid-related ED visits, inpatient hospitalizations, and opioid overdose deaths.^9^–^14^ Unintentional overdose has now surpassed motor vehicle collisions as the leading cause of injury and death in the United States for adults aged 25–64 years, and the majority of all unintentional poisonings are related to opioids.^15^, ^16^

Not surprisingly, opioid prescribing has come under increasing scrutiny in recent years including in the ED. The American College of Emergency Physicians Clinical Policy – Critical Issues in the Prescribing of Opioids for Adult Patients in the Emergency Department – states, “Although relieving pain and reducing suffering are primary emergency physician responsibilities, there is a concurrent duty to limit the personal and societal harm that can result from prescription drug misuse and abuse.“^17^ While the percentage of U.S. ED visits with opioids prescribed increased from 20.8% to 31.0% between 2001–2010, studies have shown that the majority of opioid prescriptions from the ED are a low pill count (mean of 15 tablets) and are almost exclusively (99%) immediate-release formulations and significantly less likely to be high dose or consist of a large quantity compared to those from office-based practices.^18^–^20^

Regardless, with a recent study showing that opioid-naive ED patients prescribed opioids for acute pain are at increased risk for additional opioid use at one year, the ED is an important site for the study of opioid-prescribing patterns.^21^ Adding to this body of literature, a recent article in the *New England Journal of Medicine* showed that opioid-prescribing habits vary widely between providers in the same ED and patients who receive treatment of “high-intensity” opioid prescribers had higher rates of long-term opioid use.^22^

Recent interventions for decreasing opioid prescribing have focused on prescription drug monitoring programs and creation of opioid-prescribing guidelines.^17^, ^23^–^26^ Opioid-prescribing guidelines have been shown to reduce rates of opioids prescribed for both minor and chronic complaints in acute care settings.^27^, ^28^ Most recommendations on safe opioid prescribing for the ED recommend a maximum of three-to five-day courses of opioid medications.^23^, ^29^

With the increasing prevalence of electronic medical records (EMR) and electronic order-entry systems has come an increasing interest in the ability to standardize clinical workflows in an effort to reduce medication-related errors.^30^–^33^ To date, no study has assessed the effect of default tablet quantities as part of electronic order entry on emergency physicians’ prescribing patterns. Our primary objective was to evaluate opioid-prescribing patterns before and after removal of the default quantity of 20 tablets for opioid prescriptions in the EMR.

**Population Health Research Capsule**

What do we already know about this issue?

ED opioid prescribing patterns have received increasing attention. Focus has included reducing the frequency of opioid use and alternatives to opioids for pain control.

What was the research question?

What effect would removal of a default opioid prescription quantity (20 tablets) have on ED opioid-prescribing patterns?

What was the major finding of the study?

We observed a decreased median quantity of tablets per prescription and a wider distribution in the number of tablets prescribed.

How does this improve population health?

Having providers input tablet quantities when prescribing opioids may lead to more thoughtful prescribing of opioids than having a default quantity pre-entered in the electronic medical record.

## METHODS

### Study Design

We conducted a retrospective observational study using a computer-generated dataset of consecutive patients from a single, academic, urban ED with approximately 58,000 annual visits. We followed the STROBE checklist for observational trials reporting results.^34^ This study was deemed exempt by the institutional review board as the data was originally collected for a quality improvement retrospective chart review.

### Study Setting and Population

We identified all adult patients (18 years and older) seen in a single, university-based, academic ED who were discharged home with prescriptions for tablet forms of hydrocodone and oxycodone and their acetaminophen-containing combination formulations. Patients were included only if they were discharged from the ED.

### Measurements

We examined all opioid prescriptions provided to discharged patients between 1/1/2013 and 11/3/14. The variable reviewed was opioid tablet number. We compared the quantity of tablets prescribed before and after the electronic order-entry prescription default quantity of 20 tablets was removed. This intervention was enacted on 1/17/2014. No specific messaging was given to providers to avoid influencing prescribing patterns.

### Data Analysis

We used two-sample Wilcoxon rank-sum test, two-sample test of proportions, and Pearson’s chi-squared test to compare the number of tablets prescribed before and after the removal of the default and proportion of 20 tab prescriptions. Data analysis was conducted using STATA version 14.0© (College Station, TX).

## RESULTS

A total of 4,104 adult patients received discharge prescriptions for opioids in the 54 weeks pre-intervention, and 2,464 in the 43 weeks post-intervention period. The median quantity of opioid tablets prescribed before and after removal of the default quantity was 20 (interquartile ratio [IQR] 10–20) to 15 (IQR 10–20) respectively (two sample Wilcoxon rank-sum p<0.0001). The most frequent quantity of tablets received in both groups was 20 tablets; however, the proportion of patients receiving 20 tablets reduced from 0.5 (95% confidence interval [CI] [0.48–0.52]) to 0.23 (95%CI 0.21–0.24) after default quantity removal (p<0.001) (Table 1, Figure).

## DISCUSSION

Our primary objective was to evaluate opioid-prescribing patterns after removal of default quantity of 20 tablets in the EMR. When the default quantity was in place, the majority of prescriptions provided were for this exact quantity (20 tablets), suggesting that prescribing behavior is strongly influenced by a default quantity prepopulated in the EMR. After removing the default, the number of prescriptions provided for this quantity (20) decreased, and the median number of tablets prescribed with each prescription had a statistically significant reduction. Removing the default quantity requires that physicians choose the number of tablets they will prescribe.

Had our primary objective been to achieve a more significant reduction in quantity provided, we could have changed to a smaller default value of 10 or 12 tablets and evaluated the impact of this change. However, the increased variation of tablet quantity prescriptions observed after removal of the default may reflect more appropriate prescribing patterns—a smaller quantity of analgesia tablets needed for less severe pain or pain expected to resolve quickly and greater quantities for more severe pain or pain expected to be prolonged. In many clinical scenarios it may be beneficial to avoid variation among practicing clinicians in a single ED, such as in the treatment of an acute myocardial infarction or sepsis. Having a default opioid quantity in the EMR, while demonstrated to successfully reduce variation in clinical practice patterns, may not be optimal for patient care. This would reflect a case where variation of prescribing patterns may be more appropriate than standardization. A “one-size-fits-all” approach to opioid prescribing and ignoring variable durations and severities of acute pain syndromes will predictably result in undertreatment for some patients and overtreatment for others.

The total number of prescription for opioids was also noted to have decreased during the study period. In the pre-intervention period, there were 151.6 prescriptions for opioids per 1,000 discharged adult patients compared to 106.69 per 1,000 in the post-intervention period. This may reflect random variation or purposeful decline in opioid prescribing influenced by the significant attention recently brought on by the “opioid epidemic.” The providers were not notified of removal of the default quantity; therefore, it is less likely that the intervention itself influenced the decrease in number of prescriptions. The data on prescribing patterns from the ED in recent years are limited, and it is unknown if there has been a widespread decline in prescribing patterns over this same time period.

## LIMITATIONS

This was a single-center study, potentially limiting its generalizability. However, a recent study on prescribing patterns from the ED demonstrates that the median number of pills prescribed was 15 in an observational, multi-centered cohort study across 19 U.S. EDs.^19^ These findings are similar to our department’s average tablets prescribed. Our analysis was limited to adult patients; we did not assess prescribing patterns for pediatric patients.

As a retrospective analysis, unmeasured confounders may have influenced our analysis. Factors that were not studied may have influenced opioid prescribing patterns. These include the physician’s perception of pain intensity, the age of the patient, the provider’s experience level, and the diagnosis at the time of discharge. Furthermore, it is unknown whether the increased variation post-intervention really represents true individual prescribing variation. Further evaluation would be required to analyze each individual provider’s prescribing patterns before and after the intervention to determine whether they each exhibited the same increase in variability as the entire group or if, after removal of the default quantity, each provider relied on his/her own individual default quantity for each patient regardless of painful condition.

Other potential explanations for the findings observed were not studied directly. One potential confounder is a change in the patient population or ED providers during the study period, which may have influenced prescribing habits. Comparing patient acuity in the period before and after the intervention demonstrates similar Emergency Severity Index scores and admission rates (Table 2). This suggests similar patient characteristics in the pre- and post-intervention period. The total number of Level I and II trauma activations and ED visits for adult patients was lower in the post-intervention period as expected, given the duration of the post-intervention period was shorter. Pain scores and injury severity scores may have differed and were not studied.

Although it appears that prescribing patterns may have been more appropriate after elimination of default quantity, this assumption was not directly tested. Changes in provider mix may also account for differences in opioid prescribing during the post-intervention period. Although this was not studied directly, there was minimal turnover among the provider group during the study period with a total of one hire and two departures of full-time faculty (total number of 30 faculty) during the combined time periods. Further studies would be needed to determine which factors influence physician-prescribing patterns of opioid analgesics for specific, painful conditions including analysis of pain scores.

## CONCLUSION

We demonstrated that prescription quantities for pain medications are influenced by EMR default quantities. Having a default quantity of opioid analgesics prepopulated in the EMR resulted in a large portion of patients receiving that exact quantity. Eliminating the default quantity of tablets altered prescribing patterns for clinicians, which resulted in wider variability in quantity of tablets prescribed. This may reflect more appropriate prescribing patterns for painful conditions. It is important to continue to study physician-prescribing patterns and to find strategies to prevent or reduce opioid abuse and overdose among patients while also ensuring appropriate pain control when using these high-risk medications.

## Figures and Tables

**Figure:**
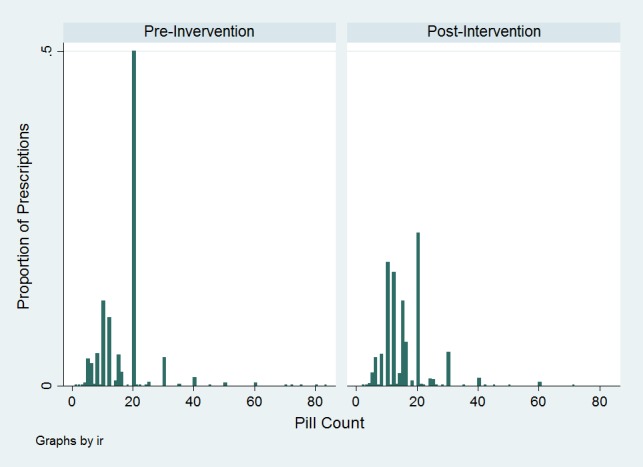
Histogram demonstrating number of tablets prescribed as a fraction of overall prescribed, opioid-containing analgesics before and after removal of default quantity of 20 tablets

**Table 1:** Number of opioid-containing analgesic prescriptions by tablet number groupings (and percentage of total prescriptions) before and after removal of default quantity of 20 tablets.

Tablet number group	Pre	Post	Total
< 20	1,723 (42.9%)	1,685 (68.4%)	3,408 (52.6%)
20	2,007 (50.0%)	562 (22.8%)	2,569 (39.7%)
> 20	284 (7.1%)	217 (8.8%)	501 (7.7%)
Total	4,014	2,464	6,478

**Table 2:** Characteristics of ED patient encounters in the time periods before and after removal of a default quantity of 20 tablets for opioid prescriptions.

Measure	Pre (1/1/13 – 1/16/14)	Post (1/17/14 – 11/3/2014)
Mean ESI Score	2.92	2.93
Median ESI Score	3	3
Admit Rate	30.6% (15,543/50,828)	29.6% (12,611/42,549)
Adult Admit Rate	33.2% (13,463/40,535)	31.8% (10,804/33,899)
Level I Traumas	298	281
Level II Traumas	1,684	1,312
Adult Patients	40,535	33,899
Total ED Visits	50,828	42,549

*ESI*, Emergency Severity Index; *ED*, emergency department
